# Impact of Glucagon-Like Peptide-1 Agonists on Hepatocellular Carcinoma Risk and Management in Type 2 Diabetes Mellitus: A Scoping Review

**DOI:** 10.7759/cureus.100252

**Published:** 2025-12-28

**Authors:** Bassam Tungekar, Evelyn C Echevarria Cruz, Jason Dodrill, Abdul Muneeb, Rachel Sanderfoot, Omar Thaj, Jeet Vaishnav, Arash Vahidi, Fadi Hindi, Rayyan Khan, Stephanie Nagy, Robin J Jacobs

**Affiliations:** 1 Dr. Kiran C. Patel College of Osteopathic Medicine, Nova Southeastern University, Fort Lauderdale, USA

**Keywords:** antidiabetic medications, cancer risk reduction, glucagon-like peptide-1 receptor agonists (glp-1ras) hepatocellular carcinoma (hcc), insulin therapy, liver disease, metformin, nonalcoholic fatty liver disease (nafld) nonalcoholic steatohepatitis (nash), type 2 diabetes mellitus (t2dm)

## Abstract

Type 2 diabetes mellitus (T2DM) is a global health burden associated with an increased risk of severe complications, including hepatocellular carcinoma (HCC). Glucagon-like peptide-1 receptor agonists (GLP-1RAs) have gained prominence in the management of T2DM due to their glucose-lowering and weight-reduction effects. Emerging evidence further suggests that GLP-1RAs may mitigate liver-related conditions such as nonalcoholic fatty liver disease and nonalcoholic steatohepatitis, both of which are major risk factors for HCC development. This scoping review aimed to summarize and map the existing evidence on the impact of GLP-1RA therapy on the risk and management of HCC in adults with T2DM. A comprehensive, systematized search was conducted across EMBASE, Ovid MEDLINE, and Web of Science using terms related to “GLP-1 receptor agonists,” “type 2 diabetes mellitus,” and “hepatocellular carcinoma.” Eligible studies included adult populations (≥18 years) with T2DM prescribed GLP-1RAs and reported outcomes specific to HCC incidence or progression. Six studies met the inclusion criteria, and most demonstrated a significantly reduced risk of HCC among patients with T2DM treated with GLP-1RAs compared with those using insulin or sulfonylureas. GLP-1RA monotherapy was generally more protective than combination therapy with insulin, whereas comparisons with metformin were inconclusive. The observed reduction in HCC risk is likely attributable to the anti-inflammatory, metabolic, and immunomodulatory effects of GLP-1RAs. Current evidence suggests that GLP-1RAs may play a protective role in reducing HCC risk among individuals with T2DM, particularly when compared with insulin-based regimens. Further longitudinal and randomized controlled trials are needed to elucidate the causal mechanisms and quantify the potential role of GLP-1RAs in reducing hepatocarcinogenesis. To our knowledge, this is the first scoping review to systematically map the literature examining GLP-1RA use and HCC risk in populations with T2DM.

## Introduction and background

Type 2 diabetes mellitus (T2DM) is a serious public health concern worldwide. Five hundred eighty-nine million adults aged 20-79 are living with diabetes worldwide, according to current data from the International Diabetes Federation. With prevalence increasing annually, it is crucial to develop effective treatment strategies to limit the progression of health complications [1]. T2DM can develop at any age and is commonly associated with insulin resistance and a degree of pancreatic beta-cell dysfunction. This leads to chronically elevated blood glucose levels [2].

The medical management of TSDM involves lifestyle modifications, such as diet, exercise, and weight control, as well as managing comorbidities and using pharmacotherapy to achieve glucose control. Metformin has long been the first-line therapy, producing moderate reductions in glucose levels and some weight loss by decreasing hepatic gluconeogenesis, improving insulin sensitivity, and enhancing peripheral glucose uptake. Glucagon-like peptide-1 receptor agonists (GLP-1RAs) are newer injectable medications used to treat T2DM for their effects on both weight reduction and glycemic control [3].

The first GLP-1RA, exenatide, was approved by the FDA in 2005 as an adjunct to diet and exercise for the treatment of T2DM [4]. Since then, several additional GLP-1RAs have been approved, including dulaglutide, liraglutide, lixisenatide, and semaglutide. All are administered via subcutaneous injection, except for an oral formulation of semaglutide [5]. GLP-1RAs act on receptors throughout the body, including the gastric mucosa, pancreatic beta cells, heart, lungs, kidneys, skin, immune cells, and hypothalamus. Their mechanism involves stimulating glucose-dependent insulin secretion, increasing GLUT4-mediated glucose uptake, slowing gastric emptying, increasing satiety, and suppressing postprandial glucagon release [6].

Although initially developed for T2DM, GLP-1RAs have gained popularity for obesity management following FDA approval of liraglutide and later semaglutide for this indication [4]. In the United States, GLP-1RA prescriptions for obesity rose from approximately 21,000 in 2019 to over 174,000 in 2023, a nearly 700% increase within four years [7].

Poorly controlled T2DM can lead to several complications, including cardiovascular disease, neuropathy, retinopathy, nephropathy, sleep apnea, delayed wound healing, hearing impairment, and liver damage [8]. Nonalcoholic fatty liver disease (NAFLD) is highly prevalent in individuals with T2DM and obesity [9]. This is due to insulin resistance in individuals with T2DM and obesity, which elevates blood levels of both insulin and glucose. The liver begins to synthesize the excess blood glucose into lipids, which promotes fat storage. NAFLD is characterized by lipid accumulation in the liver, which promotes the release of inflammatory mediators. High blood insulin levels, resulting from insulin resistance, stimulate the release of insulin-like growth factor-1 (IGF-1), promoting cell growth and division in the body, including in the liver. This overall state of inflammation and cell growth, specifically in the liver, can lead to fibrosis, DNA damage, tumor development, and progression to cancer. This is why individuals with T2DM and obesity often develop NAFLD and are at an elevated risk of progressing to nonalcoholic steatohepatitis (NASH), cirrhosis, and ultimately hepatocellular carcinoma (HCC) [9-10].

In addition to lifestyle modification, pharmacologic agents such as GLP-1RAs have been studied for their potential to prevent or improve NAFLD and NASH [9-13]. Research suggests that approximately two-thirds of patients with T2DM have NAFLD, placing them at increased risk of premature death from liver-related pathology, including HCC [14]. The chronic inflammation associated with NASH initially represents a compensatory response but, when prolonged, leads to hepatocyte injury and fibrosis, setting the stage for HCC development [15]. The GLP-1RA Wegovy (semaglutide) was approved by the FDA in August 2025 for adults with moderate-to-advanced liver scarring (fibrosis) from metabolic dysfunction-associated steatohepatitis.

Because diabetes is a significant risk factor for both NAFLD and HCC, researchers have explored the effects of antidiabetic therapies, including GLP-1RAs, on slowing and preventing liver disease progression [14-17]. Liraglutide has been shown to improve hepatic steatosis, hepatocyte ballooning, and liver enzyme levels [16]. More recent studies also highlight reductions in NAFLD incidence and hepatic transaminase elevations among patients treated with GLP-1RAs [18-19].

A 2024 review further confirmed that GLP-1RA therapy reduces liver fat and NASH, demonstrating decreases in body weight, serum biomarkers such as aspartate aminotransferase (AST) and alanine aminotransferase (ALT), and MRI-measured liver fat content in patients with NAFLD [18]. Comparative studies between GLP-1RAs and other antidiabetic agents, such as insulin, metformin, and sulfonylureas, show that GLP-1RAs are associated with reduced risks of hepatic decompensation, cirrhosis, HCC, and all-cause mortality [20-21].

Despite growing interest, a notable gap remains in research evaluating how traditional antidiabetic medications influence the life-threatening hepatic complication of HCC. Given the rising use, clinical versatility, and apparent hepatoprotective effects of GLP-1RAs, this class of medications may offer broader health benefits beyond glycemic control.

A preliminary search of the Cochrane Database of Systematic Reviews, JBI Evidence Synthesis, PubMed, and MEDLINE revealed no existing systematic or scoping reviews focused specifically on GLP-1RA use and HCC risk or management. With 589 million adults aged 20-79 currently living with diabetes and an estimated 6.7 million deaths annually from the disease, it is vital to identify therapies that not only manage glucose but also prevent downstream complications [22].

Given the sharp rise in GLP-1RA use among individuals with T2DM, a scoping review is warranted to synthesize existing evidence on their role in HCC risk reduction and disease management. This review focuses on adults aged 18 years and older with T2DM who are prescribed GLP-1RAs, excluding pediatric populations, animal studies, and cancers other than HCC.

The objective of this scoping review was to assess the extent and nature of the evidence regarding the impact of GLP-1RA therapy on HCC risk and management in individuals with T2DM. Demonstrating risk reduction or prevention of severe T2DM complications such as HCC would carry significant clinical and public health implications. The findings could help guide therapeutic decision-making toward medications that not only control glucose levels but also reduce morbidity and mortality associated with diabetes-related liver disease. The guiding research question for this review was, "How do GLP-1RAs affect the risk of developing HCC and its clinical outcomes in adult patients with T2DM?"

## Review

Methods

Search Strategy

EMBASE, Ovid Medline, and Web of Science Core Collection databases were used to perform the search. The search terms of: (GLP 1 Agonist OR GLP 1 Receptor Agonist OR Glucagon Like Peptide 1 Agonist OR Glucagon Like Peptide 1 Receptor Stimulating Agent OR Glucagon-Like Peptide-1 Receptor Agonist OR Incretin Mimetic OR Dulaglutide OR Exenatide Extended Release OR Exenatide OR Semaglutide OR Liraglutide OR Lixisenatide OR Rybelsus) AND (Non insulin dependent diabetes mellitus OR type 2 diabetes OR type 2 diabetes mellitus OR type 2 insulin independent diabetes OR insulin independent diabetes mellitus OR type II diabetes OR diabetes mellitus type II) AND (Liver cell carcinoma OR Hepatocellular carcinoma cell line OR Liver carcinogen OR Liver cancer cell line OR Liver cancer) were used to search the online databases. The search strategy, including all identified keywords and index terms, was adapted for each included database and/or information source.

A preliminary search of the Cochrane Database of Systematic Reviews, JBI Evidence Synthesis, PROSPERO, and MEDLINE found no current systematic or scoping reviews on GLP-1 peptide agonists and HCC risk and treatment management. The proposed scoping review will be conducted in accordance with the JBI methodology for scoping reviews.

Source of Evidence Selection

**Figure 1:**
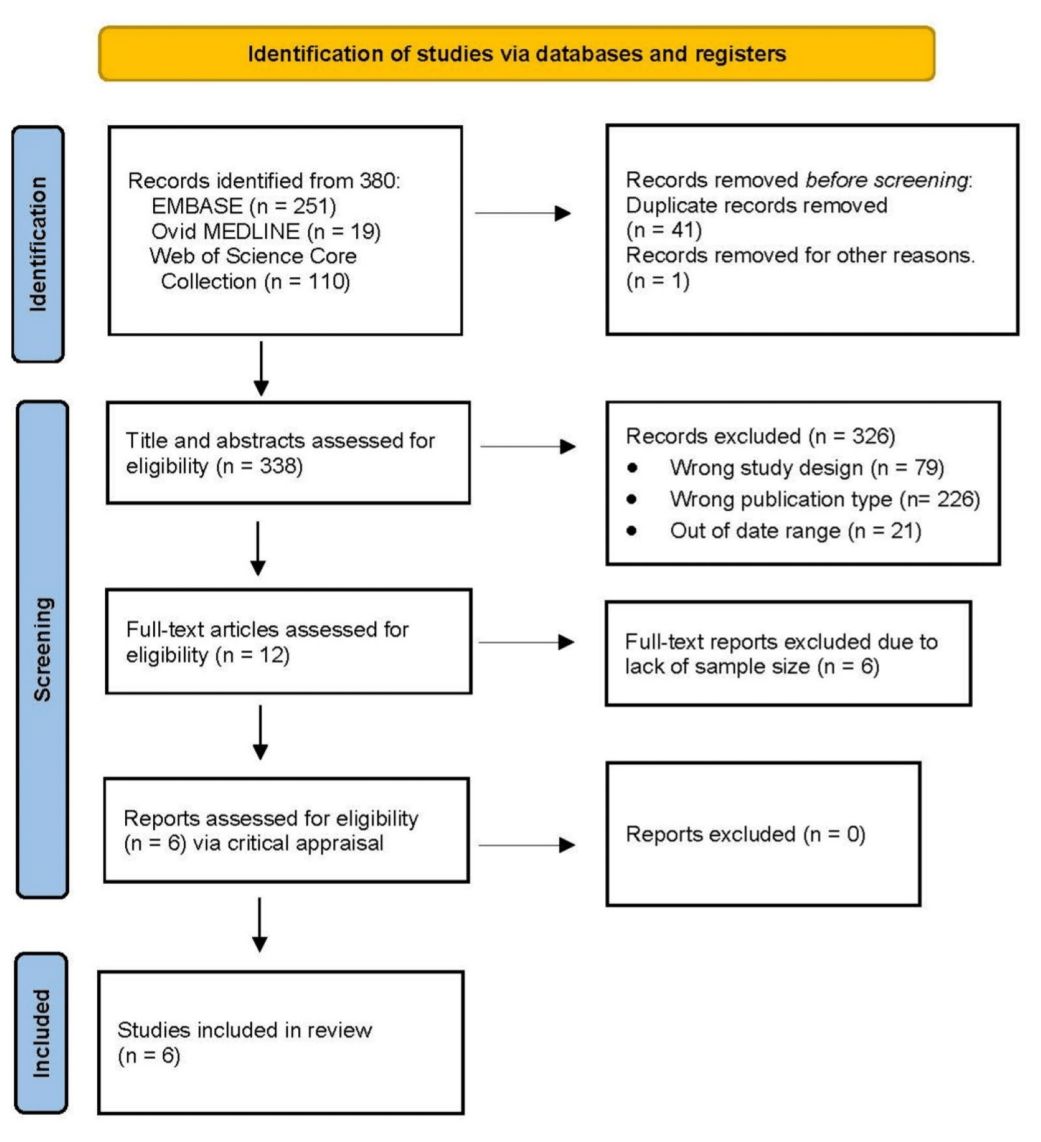
PRISMA flow diagram of the search and selection process PRISMA: Preferred Reporting Items for Systematic Reviews and Meta-Analyses

Following the search, all identified citations were collated and uploaded to a free, web-based app that accelerates the title/abstract and full-text screening stages of systematic and scoping reviews, including deduplication. Titles and abstracts were screened by the research team for eligibility based on the prespecified inclusion criteria. Potentially relevant sources will be retrieved in full, and their citation details reported. The full text of selected citations was assessed against the inclusion criteria. Reasons for excluding sources of evidence at full text that do not meet the inclusion criteria were recorded. Any disagreements among team members at each stage of the selection process were resolved through discussion or by an assigned team member serving as a tiebreaker. The results of the search and the study inclusion process are reported in Figure 1.

Data were extracted from papers included in the scoping review by two independent reviewers, with a third addressing any inconsistencies. Extracted variables were organized by title, authors, year of publication, country of origin, purpose, population and sample size, methodology, intervention type, outcome measures, relevant key findings, and study limitations. Following study selection, the quality of included studies was assessed.

Critical Appraisal of the Evidence

The Joanna Briggs Institute Critical Appraisal Tools (JBI; https://jbi.global/critical-appraisal-tools) were used to evaluate the quality of the articles. Its checklist examines factors such as research bias, overall coherence, and the essential sections that contribute to article quality. The tool assesses articles based on risk of bias: high risk (scores below 50%), moderate risk (scores between 50% and 70%), and low risk (scores above 70%).

Two team members independently read and appraised each semi-finalist article using the appropriate JBI checklist. Discrepancies were resolved through discussion until consensus was reached. All six articles were retained after detailed deliberation regarding their relevance and methodological rigor.

The population, concept, and context framework guided the search strategy. The population was defined as adults 18 and older with T2DM who were prescribed a GLP-1 agonist. The concept focused on the risk of developing HCC and its outcomes. The context encompassed any geographic or clinical setting during 2015-2025.

Inclusion and Exclusion Criteria

Studies were eligible for inclusion if they enrolled adults aged 18 years and older, as adult anatomy and physiology differ from pediatric populations, and the prevalence of both T2DM and GLP-1RA use is higher in adults. Included studies were required to focus on individuals with T2DM, given the established role of hyperglycemia and insulin resistance in hepatic fat accumulation and the subsequent increased risk of HCC.

Eligible studies also needed to evaluate exposure to GLP-1RAs, as these agents influence insulin secretion, promote weight reduction, and affect liver enzyme levels, all of which may modify hepatic cancer risk. To ensure relevance to the primary outcome of interest, only studies that explicitly included HCC as one of the cancer outcomes assessed were included. To maintain currency and consistency, only peer-reviewed studies published in English between 2015 and 2025 were considered. Eligible study designs included experimental, quasi-experimental, and randomized controlled trials, as these designs allow for a more robust evaluation of the association between GLP-1RA use and HCC risk. Studies were not excluded solely on the basis of secondary causes of NAFLD.

Studies were excluded if published before 2015, did not include patients with T2DM, were not written in English, involved participants younger than 18 years, or were conducted in animal models.

Results

This scoping review focused on six articles that evaluated the use of GLP-1RAs in patients with T2DM and liver outcomes, including the risk of developing HCC. Across the six studies, a total of 5,418,061 patients were included, with a mean age of 56.23 years.

The studies were conducted in a total of 17 countries, the majority being based in the United States (n=3), Taiwan (n=2), one study featuring Sweden, Denmark, and Norway (n=1), and a global study by Nassar et al. used a network of 119 healthcare organizations from 17 countries, primarily the USA but also including Australia, Brazil, Germany, Italy, Spain, the UK, Singapore, Malaysia, India, Taiwan, Poland, Lithuania, Bulgaria, and several others across Europe, Asia-Pacific, and Latin America. Participants in each study were being treated for T2DM.

Each of the six studies was a retrospective cohort study that utilized propensity score (PS) matching to assign 1:1 matches between study groups. PS matching was allowed for studies to assess the effects of GLP-1RA use in individuals with T2DM relative to those using other treatment modalities, including various anti-diabetes drugs, such as insulin or metformin, DPP4 inhibitors, or long-acting insulins (LAI) [20,21,23,24]. Two studies compared patients who used GLP-1RAs with those who did not [25-26]. Additionally, 1:1 PS matching within each cohort allowed these studies to reduce potential confounding variables, including patient demographics, lifestyle factors, socioeconomic status, comorbidities, and additional medication use.

The risk and management of HCC were the focus of this study; however, only two studies explicitly focused on HCC [20,24]. The four other studies took a more broad-based approach. They investigated the impact of GLP-1RAs on liver and cardiovascular diseases, obesity-associated cancers, serious liver events, and several systemic diseases [21,23,25,26]. All included studies reported outcomes related to HCC, even when HCC was not the primary focus of the investigation.

Key Findings

Association between GLP-1RA use and reduced risk of HCC: A decreased risk of HCC associated with GLP-1RA use in patients with T2DM was a central finding in four of six included studies. Specifically, when assessing systemic disease and cancer incidence, the use of GLP-1RAs versus non-use significantly reduced the risk of liver cell carcinoma in those with T2DM (p<0.001) [26].

Comparative effectiveness against other antidiabetic agents: Several studies have highlighted that GLP-1RAs reduce the risk of obesity-related cancers, including HCC, compared with insulin use [20,21,24]. However, when compared to metformin, no significant decrease in cancer risk was observed. This distinction underscores that the protective effect of GLP-1RAs may vary by comparator drug class.

Influence on obesity-associated cancers and lifestyle interventions: GLP-1RAs were shown to reduce the incidence of obesity-associated cancers, including HCC, in T2DM patients. These reductions compared favorably with those observed in the Look AHEAD trial (Action for Health in Diabetes) [21]. This suggests that GLP-1RA therapy may achieve cancer risk reductions comparable to those from major behavioral interventions.

Impact on liver-related morbidity and mortality: One study reported a 15% relative risk reduction in liver-related mortalities, attributed to decreases in both compensated and decompensated cirrhosis [23]. Another study demonstrated a 53% reduced risk of HCC with GLP-1RA use compared to LAIs. However, among patients aged ≥65 years, there was an insignificant increase in liver disease risk with GLP-1RA use versus LAIs, a finding possibly explained by wide confidence intervals [24].

Effects on hepatic decompensation and comorbidity burden: Wang et al. found that GLP-1RA use was associated with a decreased risk of hepatic decompensating events compared with six other antidiabetic medication classes. These effects were especially pronounced in patients not prescribed insulin [21]. Collectively, these studies support a hepatoprotective role of GLP-1RAs, particularly when used in place of insulin or as an adjunct to lifestyle therapy.

Studies reporting no significant reduction in HCC risk: In contrast, two of the six studies reviewed did not demonstrate a significant reduction in HCC risk associated with GLP-1RA use [23,25]. Although GLP-1RAs were associated with a lower risk of overall liver-related mortality, they did not significantly reduce HCC incidence [27]. These findings highlight some variability in the evidence base and suggest that additional controlled studies are warranted. Table 1 outlines important characteristics of the six articles, including study location, aim, methods, patient population and numbers, results, limitations, conclusions, and, if specified, the type of GLP-1RA examined.

**Table 1:** Summary of key studies evaluating GLP-1RAs and HCC HCC: hepatocellular carcinoma, GLP-1RA: glucagon-like peptide-1 receptor agonist, T2DM: type 2 diabetes mellitus, HR: hazard ratio, CI: confidence interval, SDHR: subdistribution hazard ratio, aHR: adjusted hazard ratio, CVD: cardiovascular disease, NHIRD: National Health Insurance Research Database, RCT: randomized controlled trial, LAI: long-acting insulin

Author (year)	Aim	Country/setting	Study design	Population (n, sex, mean age, follow-up)	Main findings	Limitations	Conclusions
Yen et al. (2024) [25]	Compare the risk of CVD, cirrhosis, liver-related mortality, and cardiovascular mortality between GLP-1RA users and nonusers in patients with T2DM	Taiwan (NHIRD database)	Retrospective cohort study	N = 31,156 matched pairs (F = 16,728; M = 14,428); mean age = 53.2 y; median follow up = 2 y	No significant difference in cirrhosis (aHR 1.10; 95% CI 0.88–1.37), hepatic failure (aHR 0.92; 95% CI 0.66–1.30), or HCC (aHR 0.91; 95% CI 0.59–1.40)	Missing lab data (HbA1c, glucose, lipids); limited ethnic diversity	GLP-1RA use was not linked to incident HCC but associated with lower liver-related mortality
Wang and Xu et al. (2024) [21]	Evaluate the association of GLP-1RA use with HCC risk and hepatic decompensation	United States	Retrospective cohort study	N = 809,548; mean age = 56.2 y; median follow-up = 5 y	GLP-1RA use reduced HCC risk vs insulin (HR 0.20; 95% CI 0.14–0.31) and sulfonylureas (HR 0.78; 95% CI 0.65–0.93)	Observational design; 5-year follow-up may underestimate long-term risk	GLP-1RAs were associated with reduced risk of HCC and hepatic decompensation compared with other anti-diabetic drugs
Wang et al. (2024) [20]	Compare the incident risk of obesity-associated cancers (including HCC) in patients with T2DM prescribed GLP-1RAs vs insulin or metformin	United States	Retrospective cohort study	N = 1,651,452; mean age = 59.8 y; median follow-up = 2 y	GLP-1RA use reduced HCC risk compared with insulin (HR 0.47; 95% CI 0.36–0.61)	No adherence data; medication duration not assessed	GLP-1RA use was associated with reduced risk of several obesity-related cancers, including HCC
Engström et al. (2024)[23]	Investigate the association between GLP-1RA use and serious liver events (including HCC)	Sweden, Denmark, Norway	Cohort study	N = 91,479 (GLP-1RA) vs 244,004 (DPP-4 inhibitor); mean age = 62 y; median follup = 3 y	Reduced serious liver events, but no significant difference in HCC risk (aHR 1.05; 95% CI 0.80–1.39)	Median follow-up 3 years; some lacked a confirmed T2DM diagnosis	GLP-1RA use linked to fewer liver events, but longer follow-up needed for cancer outcomes
Yang et al. (2024) [24]	Assess GLP-1RA use vs LAI and risk of cirrhosis and HCC in T2DM	Taiwan	Retrospective cohort study	N = 7,171 per group; mean age = 48.9 y; median follow-up = 1 y	GLP-1RA use decreased composite liver disease (SDHR 0.56), cirrhosis (SDHR 0.59), and HCC (SDHR 0.47)	Short follow-up (1 year); possible confounding by disease severity	GLP-1RA use was associated with lower cirrhosis and HCC risk among insulin-treated patients
Nassar et al. (2024) [26]	Evaluate whether GLP-1RA use reduces HCC and other cancers in T2DM and obesity	Multinational (TriNetX platform; 20 countries)	Retrospective cohort study	N = 854,201 per cohort; mean age = 57.3 y; median follow-up = 5 y	GLP-1RA use reduced HCC risk (risk difference: –0.002; p < 0.001) and risk of several other cancers	Observational design; causality cannot be confirmed	GLP-1RAs may lower the risk of HCC and other malignancies; RCTs needed for validation

Discussion

This scoping review synthesized current evidence on the use of GLP-1RAs in T2DM and their impact on HCC risk and management. To our knowledge, this is the first review to comprehensively examine this relationship and identify key gaps warranting further investigation.

Summary of Evidence

Among the six articles reviewed, four reported significant reductions in HCC incidence among GLP-1RA users compared with other antidiabetic therapies. At the same time, two studies showed no significant difference but still noted improvements in cirrhosis, hepatic decompensation, or overall liver-related mortality [20,21,23-26]. The most significant risk reductions were observed in comparisons with insulin-based therapies. Overall, although causation cannot currently be established, these results suggest that GLP-1RAs provide both metabolic and liver benefits, reinforcing their potential role in controlling diabetes, slowing the advancement of liver disease, and decreasing HCC risk.

Clinical Interpretation of Findings

While most studies demonstrated a statistically significant protective association between GLP-1RA use and reduced HCC risk, others reported only modest improvements in hepatic outcomes. Patients with T2DM who were most at risk of developing HCC were those treated solely with LAI monotherapy [20,21,24]. These studies still observed favorable liver-related outcomes, such as reductions in cirrhosis and hepatic decompensation, although these findings did not reach statistical significance [20,21,23-26]. The variability among results likely reflects differences in study design, comparator treatments, and follow-up duration. The study by Yen et al. used a nationwide Taiwanese database to compare GLP-1RAs users with nonusers, excluded patients with preexisting advanced liver disease, and had a median follow-up of approximately two years. While GLP-1RA use was associated with significantly lower liver-related mortality, no statistically significant difference in HCC incidence was observed, likely reflecting the shorter follow-up relative to HCC’s development period [25]. On the other hand, the Scandinavian cohort study used an active comparator, comparing GLP-1RAs with DPP-4 inhibitors, included a broader multinational population, and reported a median follow-up of 3 years. This study demonstrated a significant reduction in the incidence of severe liver events, driven primarily by a reduction in cirrhosis, but similarly did not show a statistically significant reduction in HCC [23]. Taken together, these differences suggest that insufficient follow-up duration and comparator selection likely contributed to the observed variability. As several authors noted, extended longitudinal follow-up may be necessary to capture differences in HCC incidence, given the cancer’s long latency period [23].

These findings may be clinically significant because they suggest GLP-1RAs may have benefits beyond glycemic control. Compared with other antidiabetic medications, three studies found that GLP-1RAs were associated with a significantly lower risk of HCC than LAI therapy [20,21,24]. The differences observed may reflect underlying differences in the patient population rather than direct medication effects. Patients requiring insulin typically have longer disease duration, more advanced or poorly controlled T2DM, and a greater burden of comorbidities, all of which independently are associated with increased liver disease and cancer risk. In contrast, GLP-1RAs are often initiated earlier in the disease course and improve hepatic insulin sensitivity, promote weight loss, and reduce hepatic steatosis and inflammation, collectively reducing the metabolic environment that promotes tumor genesis. As a result, comparisons between GLP-1RAs and insulin may be susceptible to confounding, which may contribute to the observed differences in HCC risk.

At the mechanistic level, exogenous insulin administration may exert mitogenic effects by activating growth factor and IGF-1 signaling pathways, thereby stimulating liver cell proliferation and decreasing apoptosis in liver tissue [28]. The absence of these proliferative signals while using GLP-1RAs could partly explain the observed variation in cancer risk in our studies. Overall, these findings suggest that the apparent advantage of GLP-1RAs over LAI may not only stem from metabolic gains but also from reduced exposure to growth-promoting signals.

Mechanisms Behind the Decreased Incidence of HCC

GLP-1RAs have demonstrated notable anti-inflammatory and immunomodulatory effects, influencing pathways that can alter tumor growth and cancer cell behavior. Specifically, liraglutide substantially decreased circulating neutrophil extracellular trap (NET) markers: myeloperoxidase, elastase, and dsDNA in Lewis lung cancer (LLC) and liver (Hepa1-6) cancer tumor‐bearing mice. NETs are key drivers of inflammatory cytokine release, including IL-6, IL-10, IL-17, and IL-21. This is in accordance with the proposed mechanisms of HCC mitigation by Nassar et al., who described cytokine downregulation leading to an anti-inflammatory microenvironment [26]. By decreasing NET formation and associated cytokine levels, liraglutide limited tumor-promoting inflammation in both lung (LLC) and liver (Hepa1-6) cancer models.

Furthermore, combining liraglutide with anti-PD-1 therapy significantly enhanced tumor suppression compared to either treatment alone, suggesting that GLP-1RAs may improve antitumor immunity by mitigating chronic inflammation that fosters cancer progression [29]. Moreover, GLP-1RAs may reduce the mean CRP concentration from 4.7 ± 0.8 mg/L to 3.2 ± 0.4 mg/L after treatment with liraglutide for a minimal duration of 6 months and a mean duration of 7.5 months (p<0.05) [30]. These findings demonstrate the possible mechanisms by which GLP-1RAs can serve as dual-purpose agents, offering metabolic benefits while attenuating inflammation that contributes to cancer progression. Interestingly, preclinical data suggest GLP-1R signaling may modulate immune activation. In rodent models of colorectal cancer, blocking GLP-1R on T-cells increased antitumor immune responses, suggesting a context-dependent role [31]. However, both clinical and metabolic investigations consistently show that GLP-1RA therapy lowers liver inflammation and the potential for cancer development, implying that the overall outcome in humans is protective for the liver rather than oncogenic.

Implications for Practice

Current diabetes management guidelines, such as those from the American Diabetes Association and the European Association for the Study of Diabetes, prioritize GLP-1RAs for patients with T2DM who have cardiovascular disease, obesity, or require weight management. Still, they do not yet consider cancer risk reduction, particularly HCC, in treatment decisions. This study suggests a possible protective role of GLP-1RAs against HCC. As evidence grows and larger clinical trials are conducted, these medications may emerge as a preferred option for patients with metabolic liver disease or those at increased risk for hepatic malignancy, including individuals with NAFLD, NASH, or cirrhosis. Clinically, this could encourage earlier use of GLP-1RAs in patients with overlapping metabolic and hepatic risk factors. It is important to note, however, that GLP-1RAs are associated with notable adverse effects, including nausea, vomiting, diarrhea, and gallstone-related complications such as cholelithiasis and acute cholecystitis [32]. From a research perspective, these findings support the need for prospective studies to confirm causality, assess drug-specific effects, and define optimal treatment duration and combinations. Incorporating liver-related outcomes into diabetes management guidelines may eventually help link metabolic and cancer-prevention strategies.

Implications for Research

Future research should address the limitations and knowledge gaps identified in this review. Since most available studies were retrospective and observational and primarily relied on large electronic health records or databases, prospective randomized controlled trials are needed to confirm the causal relationship between GLP-1RA use and reduced HCC risk. Longer follow-up durations are necessary to capture the slow progression of hepatocarcinogenesis and to understand better how chronic exposure to GLP-1RAs influences long-term liver outcomes. Additionally, as the first GLP-1RA was only approved in 2005, this therapeutic drug class remains somewhat new, and prescribing patterns have evolved rapidly, especially as these agents gained popularity for obesity and their weight loss effect. Future studies must therefore include contemporary populations reflecting this broader use.

It is equally essential that future studies aim to distinguish among individual GLP-1 compounds, doses, and treatment durations to determine whether specific agents have greater hepatoprotective effects. Mechanistic studies exploring molecular and immunologic pathways, such as the role of GLP-1RA-mediated modulation of inflammation, apoptosis, and hepatic regeneration, would help clarify whether the observed associations represent direct anticancer actions or indirect effects from metabolic improvement.

Addressing these gaps will require multicenter and likely international collaborations with standardized outcome definitions, inclusion of lifestyle and laboratory variables, and consistent stratification by liver disease stage and ethnicity. Together, such actions will provide a more complete understanding of the relationship between GLP-1RAs and HCC, ultimately guiding their future roles in both metabolic and cancer prevention.

Limitations of the Review Process

This scoping review is limited by the relatively few articles that met the eligibility criteria. A smaller number of studies can lead to significant variability across studies, including differences in study design, patient populations (NAFLD vs cirrhosis vs general T2DM), patient demographics, length of follow-up, and criteria used to define HCC. These factors can have a disproportionate impact on the overall interpretation of the results, therefore restricting the ability to draw standardized conclusions.

Limitations of the Articles in the Review

Each included study had methodological limitations that warrant consideration. All of the included studies were retrospective and observational, precluding causal inference, being prone to bias and incompleteness, and failing to fully eliminate confounding effects despite study-level adjustments to mitigate this [20-21,23-26]. Randomized controlled trials would be better suited to explore the causal nature of the aim of this scoping review. GLP-1RAs are a broad category; for example, liraglutide was the most used GLP-1RA in Engström et al., so the results are more specific to that drug [25]. Therefore, more extensive research is needed into the separate GLP-1RAs and their respective impacts on risk and management of HCC.

Additionally, some cohorts were predominantly of Taiwanese or Scandinavian ethnicity, restricting the generalizability of findings to other ethnic groups [23-25]. Some studies relied on electronic health record prescriptions without adherence or duration data, which may introduce exposure misclassification [20-21].

When examining individual studies, Engström et al. did not explicitly confirm T2DM diagnoses because primary-care data were unavailable, allowing diagnostic uncertainty and confounding by indication [23]. Yang et al. mainly focused on patients with advanced or poorly controlled diabetes, which could have introduced potential confounding by disease severity [24]. Similarly, Yen et al. used a national insurance database that did not track lifestyle and metrics such as smoking, diet, physical activity, HbA1c, lipids, liver function, imaging, and histopathologic data [25].

Notably, GLP-1RAs represent a diverse pharmacologic class, yet liraglutide was the predominant agent used in the study by Engström et al., making the findings more specific to that drug [23]. Moreover, while follow-up times were mostly a few years, the longest follow-up period was 15 years, which may still not entirely capture the long-term development of HCC [21].

Together, these limitations highlight substantial heterogeneity in study design, population characteristics, duration of follow-up, and data completeness across the studies. These factors demonstrate the need for long-term, well-controlled prospective studies to clarify whether GLP-1RAs do have a direct protective effect against HCC and associated liver complications in patients with T2DM. Despite these limitations, this review provides one of the first synthesized summaries of the association between GLP-1RA use and HCC risk.

## Conclusions

This scoping review indicates that GLP-1RAs may confer protective benefits against HCC in individuals with T2DM, particularly when compared with agents such as DPP-4 inhibitors and LAI. Although findings related to metformin remain inconclusive, emerging evidence supports potential anti-inflammatory, metabolic, and immunomodulatory mechanisms through which GLP-1RAs may reduce liver cancer risk. Notably, the protective effect appears more pronounced with GLP-1RA monotherapy than with combination therapy involving insulin, underscoring key clinical considerations for treatment selection. Given the growing global prevalence of T2DM and its progression to liver-related complications, GLP-1RAs show promise as dual-purpose agents that may offer both metabolic and hepatoprotective advantages. However, the existing evidence base is limited by heterogeneity in study design, follow-up duration, and comparator treatments. Robust prospective and randomized controlled trials are needed to confirm causality, delineate drug-specific effects, and clarify the long-term impact of GLP-1RA therapy on HCC prevention and management. As worldwide use of GLP-1RAs continues to expand, recognizing and further exploring their potential role in reducing HCC risk could have meaningful implications for both metabolic and oncologic care.
